# Preparation of a chloride salt of covalently modified isoniazid

**DOI:** 10.1107/S2056989026004809

**Published:** 2026-05-29

**Authors:** Rolivhuwa Mahwasane, Mark G. Smith

**Affiliations:** a University of South Africa, Chemistry Department, Unisa Science Campus, 28 Pioneer Avenue, Florida, Roodepoort, Gauteng, South Africa; Venezuelan Institute of Scientific Research, Venezuela

**Keywords:** crystal structure, isoniazid, chloride salt, covalent modification, layering method

## Abstract

A novel covalently modified isoniazid salt {4-[*N*′-(propan-2-yl­idene)hydrazinecarbon­yl]pyridin-1-ium chloride}, C_9_H_12_N_3_O^+^·Cl^−^, was synthesized using a slow diffusion layering technique under ambient conditions. Single-crystal X-ray diffraction (SC-XRD) analysis revealed that the compound crystallizes in the triclinic space group *P*1. The crystal structure features prominent N—H⋯Cl hydrogen bonds, indicating strong inter­molecular inter­actions between the chloride ion and the modified isoniazid framework.

## Abstract 2

A novel covalently modified isoniazid salt {4-[*N*′-(propan-2-yl­idene)hydrazinecarbon­yl]pyridin-1-ium chloride}, C_9_H_12_N_3_O^+^·Cl^−^, was synthesized using a slow diffusion layering technique under ambient conditions. The crystal structure features prominent N—H⋯Cl hydrogen bonds, indicating strong inter­molecular inter­actions between the chloride ion and the modified isoniazid framework.

## 1. Chemical context

Isoniazid (pyridine-4-carb­oxy­lic acid hydrazide) is a well-established first-line anti­tubercular drug that remains a cornerstone of combination therapy for tuberculosis worldwide (Hegde *et al.*, 2021 ▸). Owing to its clinical significance, isoniazid has been the subject of extensive structural modification efforts aimed at improving its physicochemical and pharmacological properties (Setshedi *et al.*, 2022 ▸; Smith & Lemmerer, 2018 ▸). One such strategy involves condensation of the hydrazide moiety with a carbonyl compound, yielding a hydrazone derivative while preserving the integrity of the pyridine ring (Lemmerer, 2012 ▸). This covalent transformation introduces an imine C=N double bond adjacent to the aromatic system, enhancing mol­ecular rigidity and modulating the electronic environment of the hydrazide functionality (Lemmerer, 2012 ▸). Although the pyridine nitro­gen remains chemically unaltered, it frequently participates in strong hydrogen bonding, which plays a critical role in stabilizing the crystal structure (Aakoröy *et al.*, 2007 ▸; Setshedi *et al.*, 2021 ▸). These directional inter­actions often organize the mol­ecular components into extended supra­molecular chains or layered assemblies, defining the overall packing architecture. The neutral hydrazone compound was first synthesized by Wang *et al.* (2008 ▸), and then by Lemmerer (2012 ▸); however, its isolation and crystallographic characterization as a salt have not previously been reported. Covalent modification of isoniazid derivatives often leads to improved activity against multi-drug resistant tuberculosis (Hearn *et al.*, 2004 ▸; Setshedi & Smith, 2021 ▸) and their crystal structures provide valuable insights into the role of ionic inter­actions and hydrogen-bonding networks in consolidating the solid state (Scheepers & Lemmerer, 2023 ▸).
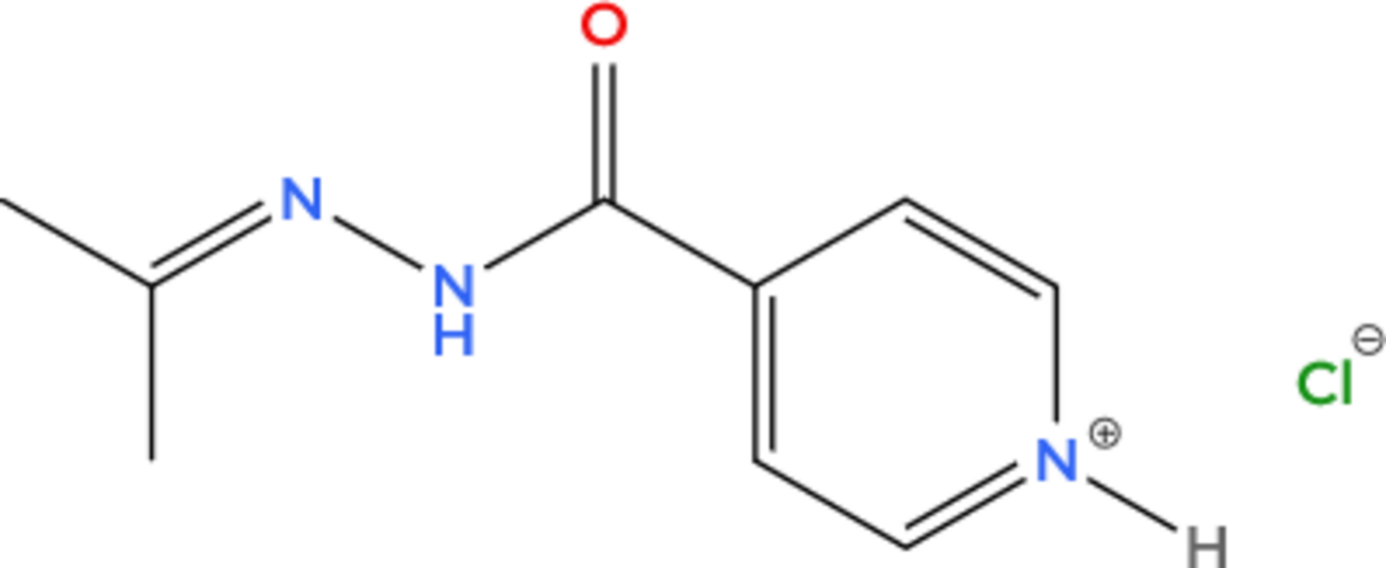


## 2. Structural commentary

**Figure 1:**
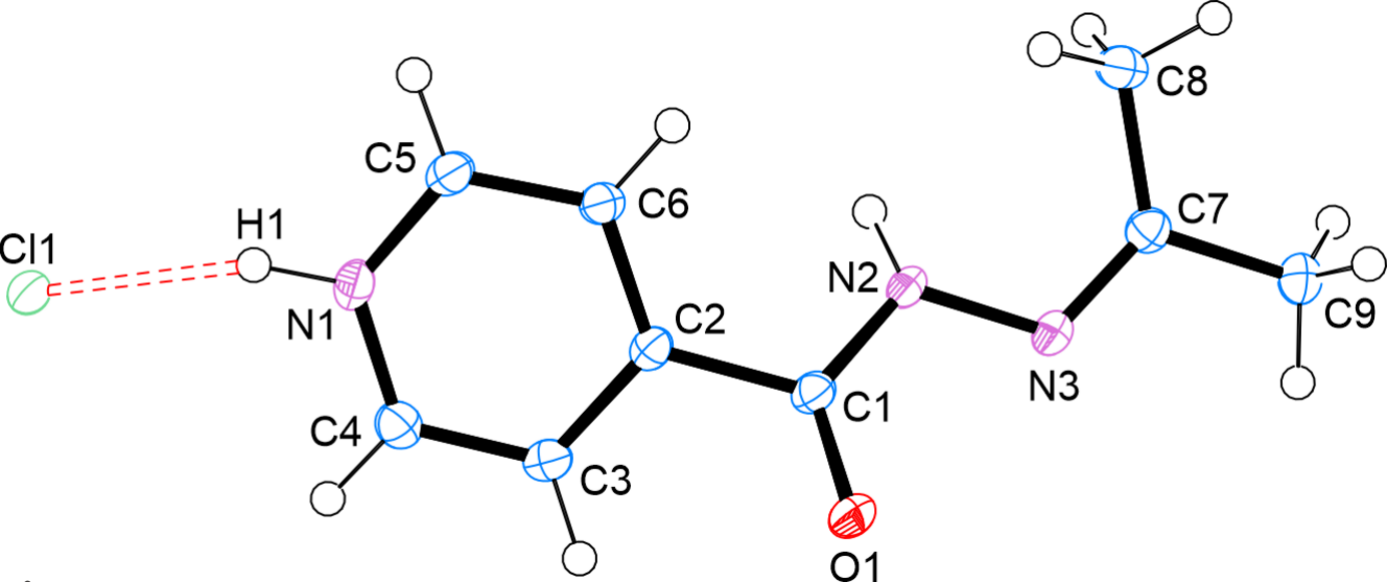
The asymmetric unit of the chloride salt of covalently modified isoniazid with displacement ellipsoids drawn at the 50% probability level.

The asymmetric unit of the title compound comprises one mol­ecule of the covalently modified isoniazid hydrazone, namely *N′*-(2-propan­ylidene)isonicotinohydrazide and one chloride ion (Fig. 1 ▸). The hydrazone moiety adopts an extended conformation, with the imine C=N double bond in the (*E*)-configuration, consistent with previously reported structures of neutral analogues. The chloride ion is located in close proximity to the hydrazide N—H donor, forming a strong N—H⋯Cl hydrogen bond that anchors the ionic framework (Table 1 ▸). All bond lengths and angles fall within expected ranges for hydrazone derivatives. The C=N bond measures 1.2835 (15) Å, confirming its double-bond character, while the N—N and C=O distances are consistent with typical hydrazide geometry.

**Figure 2:**
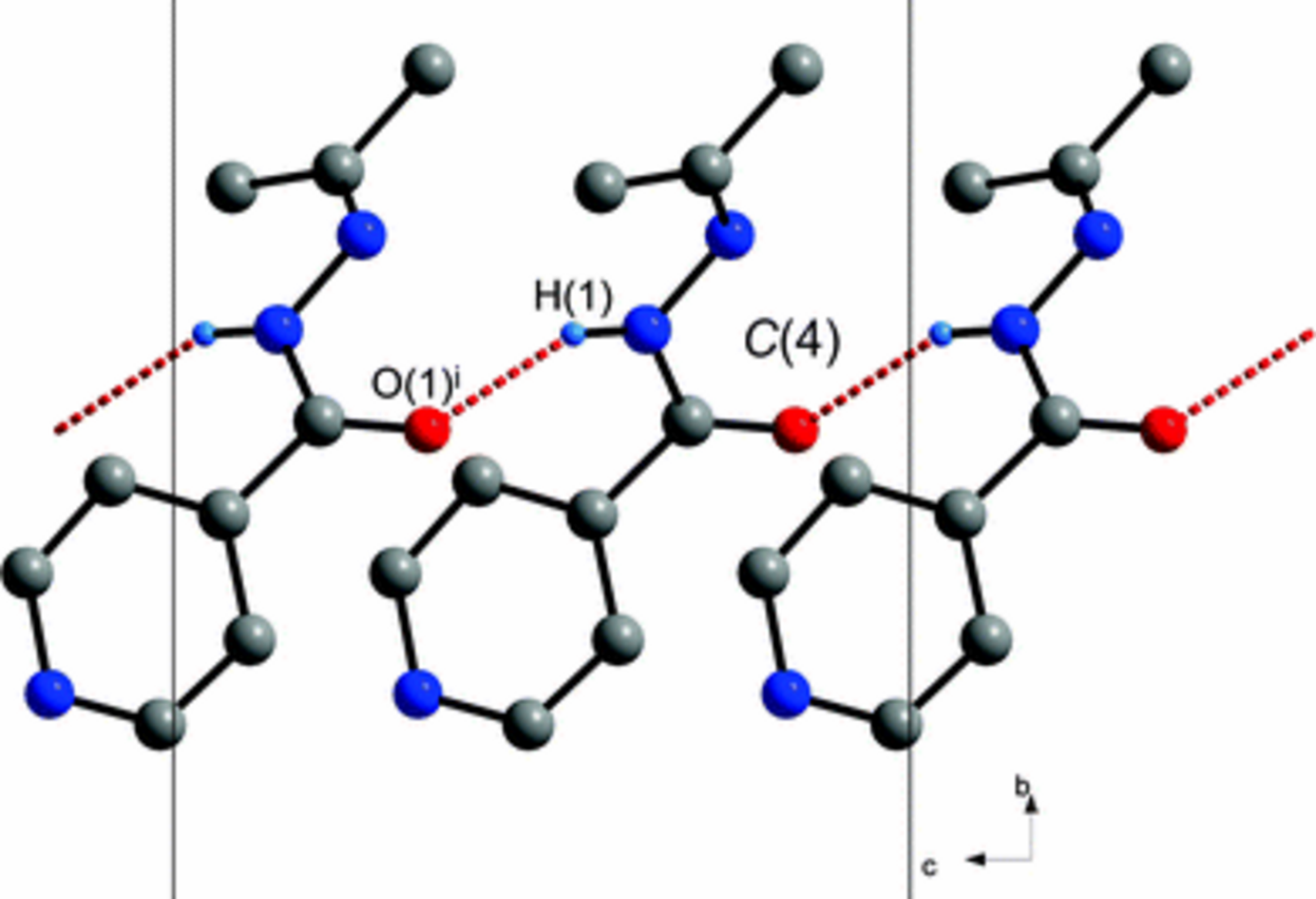
The crystal structure of *N*′-(2-propanyl­idene)isonicotinohydrazide. Reproduced directly from Lemmerer (2012 ▸).

**Figure 3:**
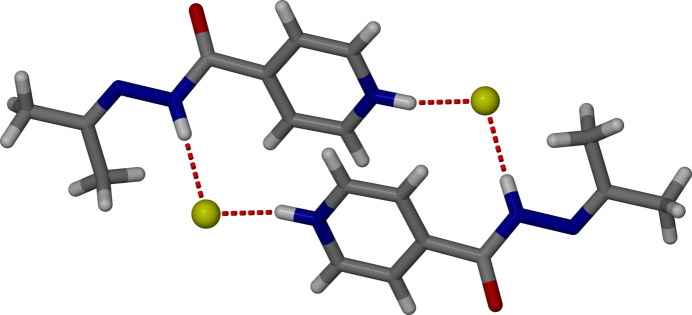
The four-membered hydrogen-bonded ring motif of the chloride salt.

In the free base, *N′*-(2-propanyl­idene)isonicotinohydrazide, the crystal structure is consolidated by hydrogen bonding between the carbonyl oxygen acceptor and the amide hydrogen donor (Lemmerer, 2012 ▸; Fig. 2 ▸). In contrast, in the chloride salt reported in this study, the amide hydrogen donor inter­acts with a chloride ion, forming a chloride bridge. The chloride ion is further hydrogen bonded to the protonated (aminium) nitro­gen of the pyridine ring, resulting in a four-membered hydrogen-bonded ring motif (Fig. 3 ▸).

## 3. Supra­molecular features

**Figure 4:**
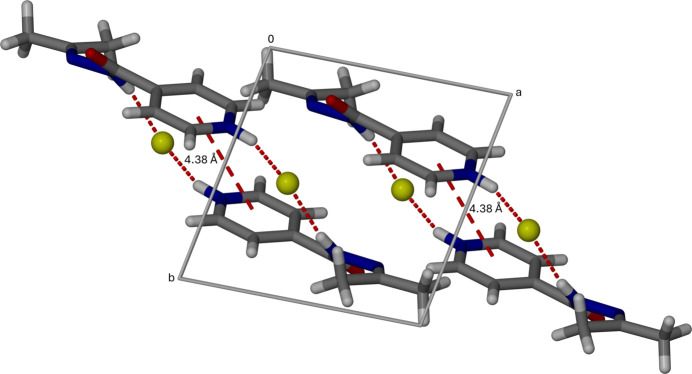
Crystal packing diagram of the chloride salt of covalently modified isoniazid, viewed along the *c*-axis, highlighting π–π stacking inter­actions between adjacent pyridine rings.

The packing arrangement of the title compound is illustrated in Fig. 4 ▸. In the crystal, the chloride anion forms a strong, directional N—H⋯Cl hydrogen bond with the hydrazide N—H donor, with an H⋯Cl distance of 2.1 Å and an N—H⋯Cl angle of 175° (Table 1 ▸). These inter­actions link cations and anions into one-dimensional chains along the *c*-axis direction, which are further connected into two-dimensional layers through weaker C—H⋯Cl inter­actions (Table 1 ▸) and van der Waals contacts. Adjacent pyridine rings are approximately parallel, with a centroid–centroid distance of 4.3823 (7) Å, indicative of weak long-range π–π inter­actions that may contribute subtly to the packing cohesion, also seen in Fig. 4 ▸. Overall, the supra­molecular assembly is dominated by the strong N—H⋯Cl hydrogen bonding, with secondary inter­actions supporting the layered crystal architecture.

## 4. Database survey

In this paper, we report the chloride salt of *N′*-(2-propanyl­idene)isonicotinohydrazide, obtained via reaction with iso­propanol. A search of the Cambridge Structural Database (CSD, Version 2025.1; Groom *et al.*, 2016 ▸) identified 15 crystal structures of *N′*-(2-propanyl­idene)isonicotinohydrazide. The structure of the free base has previously been published by Wang *et al.* (2008 ▸, refcode ROFCIZ) and Lemmerer *et al.* (2012 ▸, refcode ROFCIZ01). Notably, all 15 covalently modified structures were prepared from the reaction of isoniazid with acetone. In contrast, the salt presented here was obtained from isoniazid and iso­propanol in the presence of an iron catalyst. Inter­estingly, aside from the two free-base structures mentioned above and a hydrate reported by Álvarez-Vidaurre *et al.* (2021 ▸, refcode UQEJEI), all acetone-derived isoniazid derivatives crystallized as co-crystals. By contrast, synthesis from iso­propanol, as reported here, yielded a salt.

## 5. Synthesis and crystallization

All reagents were commercially sourced and used without further purification. To synthesize the title compound, FeCl_3_ (162.21 mg, 1.00 mmol) was dissolved in 3 ml of DMSO by stirring at room temperature for 10 minutes. Once fully dissolved, isoniazid (INH) (137.1 mg, 1.00 mmol) was added with continuous stirring, followed by the addition of three drops of concentrated hydro­chloric acid. The reaction mixture was stirred for a further 20 minutes. The resulting solution was carefully layered with 4 ml of isopropanol and left undisturbed at room temperature (±298.15 K) for two weeks. This procedure yielded two distinct crystalline forms: green crystals of the iron complex and colourless crystals of the corresponding salt.

## 6. Refinement

Crystal data, data collection, and structure refinement details are summarized in Table 2 ▸. Carbon-bound hydrogen atoms were first located in the difference Fourier map, then positioned geometrically and refined using a riding model, with isotropic displacement parameters set to 1.2 times those of their parent carbon atoms. The coordinates of the nitro­gen-bound hydrogen atom involved in hydrogen bonding inter­actions were refined freely, with isotropic displacement parameters set to 1.5 times those of the parent nitro­gen atom.

## Supplementary Material

Crystal structure: contains datablock(s) I, global. DOI: 10.1107/S2056989026004809/zn2045sup1.cif

Structure factors: contains datablock(s) I. DOI: 10.1107/S2056989026004809/zn2045Isup2.hkl

Supporting information file. DOI: 10.1107/S2056989026004809/zn2045Isup3.mol

Supporting information file. DOI: 10.1107/S2056989026004809/zn2045Isup4.cml

CCDC reference: 2488835

Additional supporting information:  crystallographic information; 3D view; checkCIF report

## Figures and Tables

**Table 1:** Hydrogen-bond geometry (Å, °)

*D*—H⋯*A*	*D*—H	H⋯*A*	*D*⋯*A*	*D*—H⋯*A*
N1—H1⋯Cl1	0.920 (16)	2.137 (16)	3.0120 (10)	158.8 (14)
N2—H2⋯Cl1^i^	0.897 (15)	2.314 (16)	3.2012 (10)	170.1 (14)
C3—H3⋯Cl1^ii^	0.95	2.75	3.6711 (12)	163
C4—H4⋯Cl1^iii^	0.95	2.74	3.5670 (12)	146
C5—H5⋯O1^iv^	0.95	2.45	3.1487 (15)	131
C5—H5⋯N3^iv^	0.95	2.38	3.2936 (15)	161

Symmetry codes: (i) 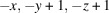; (ii) 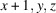; (iii) 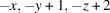; (iv) 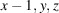.

**Table 2:** Experimental details

Crystal data	
Chemical formula	C_9_H_12_N_3_O^+^·Cl^−^
*M*_r_	213.67
Crystal system, space group	Triclinic, *P* 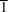
Temperature (K)	100
*a*, *b*, *c* (Å)	7.6446 (2), 7.8650 (3), 8.7236 (3)
α, β, γ (°)	100.488 (1), 91.053 (1), 100.038 (1)
*V* (Å^3^)	507.17 (3)
*Z*	2
Radiation type	Mo *K*α
μ (mm^−1^)	0.35
Crystal size (mm)	0.58 × 0.30 × 0.17

Data collection	
Diffractometer	Bruker APEXII CCD
Absorption correction	–
No. of measured, independent and observed [*I* > 2σ(*I*)] reflections	28203, 2332, 2258
*R*_int_	0.029
(sin θ/λ)_max_ (Å^−1^)	0.650

Refinement	
*R*[*F*^2^ > 2σ(*F*^2^)], *wR*(*F*^2^), *S*	0.026, 0.076, 1.11
No. of reflections	2324
No. of parameters	135
H-atom treatment	H atoms treated by a mixture of independent and constrained refinement
Δρ_max_, Δρ_min_ (e Å^−3^)	0.38, −0.21

Computer programs: *APEX3*, *SAINT-Plus* and *XPREP* (Bruker 2016 ▸), *OLEX2* (Dolomanov *et al.*, 2019 ▸), *SHELXT2014* (Sheldrick, 2015*a* ▸), *SHELXL2014/7* (Sheldrick, 2015*b* ▸), *ORTEP-3 for Windows* and *WinGX* publication routines (Farrugia, 2012 ▸), *Mercury* (Macrae *et al.*, 2020 ▸) and *PLATON* (Spek, 2020 ▸).

## supplementary crystallographic information

### 4-[N'-(Propan-2-ylidene)hydrazinecarbonyl]pyridin-1-ium chloride Crystal data

**Table:**

C_9_H_12_N_3_O^+^·Cl^−^	*Z* = 2
*M**_r_* = 213.67	*F*(000) = 224
Triclinic, *P*1	*D*_x_ = 1.399 Mg m^−^^3^
*a* = 7.6446 (2) Å	Mo *K*α radiation, λ = 0.71073 Å
*b* = 7.8650 (3) Å	Cell parameters from 9473 reflections
*c* = 8.7236 (3) Å	θ = 2.4–27.5°
α = 100.488 (1)°	µ = 0.35 mm^−^^1^
β = 91.053 (1)°	*T* = 100 K
γ = 100.038 (1)°	Block, colourless
*V* = 507.17 (3) Å^3^	0.57 × 0.30 × 0.17 mm

### 4-[N'-(Propan-2-ylidene)hydrazinecarbonyl]pyridin-1-ium chloride Data collection

**Table:**

Bruker APEXII CCD diffractometer	*R*_int_ = 0.029
φ and ω scans	θ_max_ = 27.5°, θ_min_ = 2.4°
28203 measured reflections	*h* = −9→9
2332 independent reflections	*k* = −10→10
2258 reflections with *I* > 2σ(*I*)	*l* = −11→11

### 4-[N'-(Propan-2-ylidene)hydrazinecarbonyl]pyridin-1-ium chloride Refinement

**Table:**

Refinement on *F*^2^	0 constraints
Least-squares matrix: full	Hydrogen site location: mixed
*R*[*F*^2^ > 2σ(*F*^2^)] = 0.026	H atoms treated by a mixture of independent and constrained refinement
*wR*(*F*^2^) = 0.076	*w* = 1/[σ^2^(*F*_o_^2^) + (0.0365*P*)^2^ + 0.2176*P*] where *P* = (*F*_o_^2^ + 2*F*_c_^2^)/3
*S* = 1.11	(Δ/σ)_max_ = 0.001
2324 reflections	Δρ_max_ = 0.38 e Å^−^^3^
135 parameters	Δρ_min_ = −0.21 e Å^−^^3^
0 restraints	

### 4-[N'-(Propan-2-ylidene)hydrazinecarbonyl]pyridin-1-ium chloride Special details

**Table:**

Geometry. All esds (except the esd in the dihedral angle between two l.s. planes) are estimated using the full covariance matrix. The cell esds are taken into account individually in the estimation of esds in distances, angles and torsion angles; correlations between esds in cell parameters are only used when they are defined by crystal symmetry. An approximate (isotropic) treatment of cell esds is used for estimating esds involving l.s. planes.
Refinement. The crystal structure was solved with *OLEX2* (Dolomanov *et al.*, 2019) by direct methods using SHELXT (Sheldrick, 2015). Non-hydrogen atoms were initially refined isotropically, followed by anisotropic refinement using full-matrix least-squares calculations based on F^2^ with *SHELXL*.

### 4-[N'-(Propan-2-ylidene)hydrazinecarbonyl]pyridin-1-ium chloride Fractional atomic coordinates and isotropic or equivalent isotropic displacement parameters (Å^2^)

**Table:**

	*x*	*y*	*z*	*U*_iso_*/*U*_eq_	
C1	0.55980 (15)	0.78876 (14)	0.54588 (13)	0.0168 (2)	
C3	0.35037 (15)	0.65162 (15)	0.72127 (13)	0.0179 (2)	
H3	0.449994	0.624807	0.772559	0.022*	
C4	0.18139 (16)	0.60644 (15)	0.77089 (13)	0.0193 (2)	
H4	0.163402	0.546093	0.855847	0.023*	
C8	0.62897 (16)	0.87847 (17)	0.10016 (14)	0.0227 (2)	
H8A	0.569495	0.768289	0.034188	0.034*	
H8B	0.683094	0.959495	0.034062	0.034*	
H8C	0.541702	0.931803	0.164363	0.034*	
C9	0.95546 (16)	0.85452 (17)	0.14704 (14)	0.0224 (2)	
H9A	1.006503	0.978093	0.148385	0.034*	
H9B	0.951451	0.787694	0.040233	0.034*	
H9C	1.029234	0.806303	0.215253	0.034*	
C6	0.22523 (15)	0.77914 (15)	0.52266 (13)	0.0169 (2)	
H6	0.239081	0.838311	0.436726	0.02*	
C2	0.37274 (14)	0.73748 (14)	0.59433 (12)	0.0156 (2)	
C5	0.05897 (15)	0.73335 (15)	0.57808 (13)	0.0184 (2)	
H5	−0.042623	0.761916	0.531327	0.022*	
C7	0.77068 (15)	0.84150 (14)	0.20401 (13)	0.0176 (2)	
N2	0.57561 (12)	0.78193 (13)	0.39063 (11)	0.0170 (2)	
H2	0.485 (2)	0.724 (2)	0.3240 (18)	0.02*	
N1	0.04194 (13)	0.64795 (13)	0.69882 (11)	0.0182 (2)	
H1	−0.069 (2)	0.613 (2)	0.7336 (18)	0.022*	
N3	0.75011 (12)	0.80011 (13)	0.33906 (11)	0.0180 (2)	
O1	0.68331 (11)	0.83507 (12)	0.64431 (10)	0.02301 (19)	
Cl1	−0.27331 (3)	0.46775 (3)	0.84678 (3)	0.01841 (10)	

### 4-[N'-(Propan-2-ylidene)hydrazinecarbonyl]pyridin-1-ium chloride Atomic displacement parameters (Å^2^)

**Table:**

	*U*^11^	*U*^22^	*U*^33^	*U*^12^	*U*^13^	*U*^23^
C1	0.0136 (5)	0.0184 (5)	0.0191 (5)	0.0041 (4)	−0.0001 (4)	0.0044 (4)
C3	0.0169 (5)	0.0193 (5)	0.0177 (5)	0.0048 (4)	−0.0017 (4)	0.0027 (4)
C4	0.0206 (6)	0.0202 (5)	0.0172 (5)	0.0034 (4)	0.0014 (4)	0.0037 (4)
C8	0.0226 (6)	0.0286 (6)	0.0193 (5)	0.0075 (5)	−0.0001 (4)	0.0077 (5)
C9	0.0183 (6)	0.0281 (6)	0.0211 (5)	0.0035 (5)	0.0040 (4)	0.0060 (5)
C6	0.0155 (5)	0.0193 (5)	0.0160 (5)	0.0040 (4)	−0.0004 (4)	0.0033 (4)
C2	0.0132 (5)	0.0166 (5)	0.0160 (5)	0.0026 (4)	0.0001 (4)	0.0004 (4)
C5	0.0151 (5)	0.0217 (5)	0.0179 (5)	0.0048 (4)	−0.0012 (4)	0.0012 (4)
C7	0.0169 (5)	0.0177 (5)	0.0176 (5)	0.0031 (4)	0.0006 (4)	0.0021 (4)
N2	0.0105 (4)	0.0229 (5)	0.0175 (5)	0.0025 (4)	0.0001 (3)	0.0038 (4)
N1	0.0137 (5)	0.0207 (5)	0.0186 (5)	0.0014 (4)	0.0029 (4)	0.0011 (4)
N3	0.0124 (4)	0.0224 (5)	0.0195 (5)	0.0034 (4)	0.0015 (3)	0.0046 (4)
O1	0.0136 (4)	0.0350 (5)	0.0193 (4)	0.0012 (3)	−0.0022 (3)	0.0054 (3)
Cl1	0.01409 (14)	0.02298 (15)	0.01776 (15)	0.00193 (10)	0.00002 (9)	0.00423 (10)

### 4-[N'-(Propan-2-ylidene)hydrazinecarbonyl]pyridin-1-ium chloride Geometric parameters (Å, º)

**Table:**

C1—C2	1.5065 (15)	C9—H9B	0.98
C1—N2	1.3541 (15)	C9—H9C	0.98
C1—O1	1.2239 (14)	C9—C7	1.4989 (16)
C3—H3	0.95	C6—H6	0.95
C3—C4	1.3780 (16)	C6—C2	1.3954 (15)
C3—C2	1.3966 (16)	C6—C5	1.3804 (16)
C4—H4	0.95	C5—H5	0.95
C4—N1	1.3453 (15)	C5—N1	1.3452 (15)
C8—H8A	0.98	C7—N3	1.2835 (15)
C8—H8B	0.98	N2—H2	0.896 (16)
C8—H8C	0.98	N2—N3	1.4076 (13)
C8—C7	1.4995 (16)	N1—H1	0.918 (16)
C9—H9A	0.98		
			
N2—C1—C2	114.95 (9)	C7—C9—H9C	109.5
O1—C1—C2	120.18 (10)	C2—C6—H6	120.4
O1—C1—N2	124.87 (10)	C5—C6—H6	120.4
C4—C3—H3	120.6	C5—C6—C2	119.22 (10)
C4—C3—C2	118.90 (10)	C3—C2—C1	117.40 (10)
C2—C3—H3	120.6	C6—C2—C1	122.83 (10)
C3—C4—H4	120.1	C6—C2—C3	119.70 (10)
N1—C4—C3	119.88 (11)	C6—C5—H5	120.3
N1—C4—H4	120.1	N1—C5—C6	119.47 (10)
H8A—C8—H8B	109.5	N1—C5—H5	120.3
H8A—C8—H8C	109.5	C9—C7—C8	117.72 (10)
H8B—C8—H8C	109.5	N3—C7—C8	126.34 (10)
C7—C8—H8A	109.5	N3—C7—C9	115.94 (10)
C7—C8—H8B	109.5	C1—N2—H2	119.5 (10)
C7—C8—H8C	109.5	C1—N2—N3	116.06 (9)
H9A—C9—H9B	109.5	N3—N2—H2	119.3 (10)
H9A—C9—H9C	109.5	C4—N1—H1	117.2 (10)
H9B—C9—H9C	109.5	C5—N1—C4	122.81 (10)
C7—C9—H9A	109.5	C5—N1—H1	120.0 (10)
C7—C9—H9B	109.5	C7—N3—N2	116.08 (9)
			
C1—N2—N3—C7	−161.70 (10)	C2—C6—C5—N1	−0.70 (16)
C3—C4—N1—C5	−0.05 (17)	C5—C6—C2—C1	−177.47 (10)
C4—C3—C2—C1	178.54 (10)	C5—C6—C2—C3	−0.47 (16)
C4—C3—C2—C6	1.38 (16)	N2—C1—C2—C3	146.32 (10)
C8—C7—N3—N2	2.47 (17)	N2—C1—C2—C6	−36.62 (15)
C9—C7—N3—N2	−177.92 (9)	O1—C1—C2—C3	−34.86 (15)
C6—C5—N1—C4	0.99 (17)	O1—C1—C2—C6	142.20 (12)
C2—C1—N2—N3	−169.69 (9)	O1—C1—N2—N3	11.56 (17)
C2—C3—C4—N1	−1.14 (17)		

### 4-[N'-(Propan-2-ylidene)hydrazinecarbonyl]pyridin-1-ium chloride Hydrogen-bond geometry (Å, º)

**Table:**

*D*—H···*A*	*D*—H	H···*A*	*D*···*A*	*D*—H···*A*
N1—H1···Cl1	0.920 (16)	2.137 (16)	3.0120 (10)	158.8 (14)
N2—H2···Cl1^i^	0.897 (15)	2.314 (16)	3.2012 (10)	170.1 (14)
C3—H3···Cl1^ii^	0.95	2.75	3.6711 (12)	163
C4—H4···Cl1^iii^	0.95	2.74	3.5670 (12)	146
C5—H5···O1^iv^	0.95	2.45	3.1487 (15)	131
C5—H5···N3^iv^	0.95	2.38	3.2936 (15)	161

Symmetry codes: (i) −*x*, −*y*+1, −*z*+1; (ii) *x*+1, *y*, *z*; (iii) −*x*, −*y*+1, −*z*+2; (iv) *x*−1, *y*, *z*.
